# Supplementary material for: Chromosome-scale assemblies of the male and female Populus euphratica genomes reveal the molecular basis of sex determination and sexual dimorphism
Source: Commun Biol. 2022 Nov 4;5:1186. doi: 10.1038/s42003-022-04145-7 (PMC9636151; doi:10.1038/s42003-022-04145-7)
Supplement: Supplementary file 2 — Supplementary Information [file 42003_2022_4145_MOESM2_ESM.pdf]

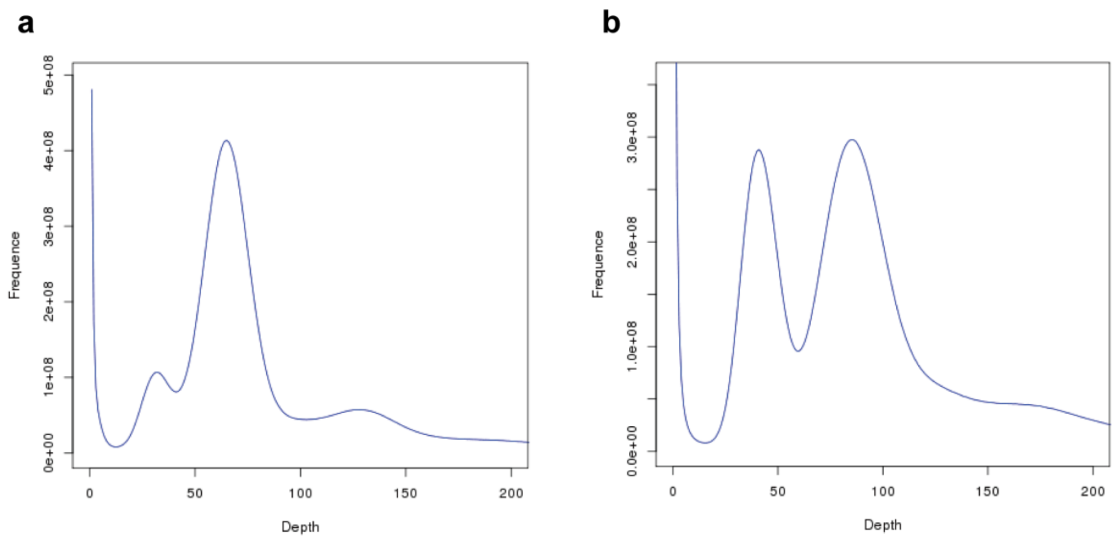

Supplementary Figure 1. The k-mer analysis (k=17) of *Populus euphratica* genome. 17-mer coverage distribution in the *P. euphratica* genomes of female TF1-136 (**a**) and male TM7 (**b**) individuals. When k-mer = 17, a frequency peak value at 64 and 84 for the female (**a**) or male (**b**) genome, respectively, was used to estimate genome size. The x-axis shows the coverage depth of 17-mers; the y-axis shows the coverage frequency of 17-mers.

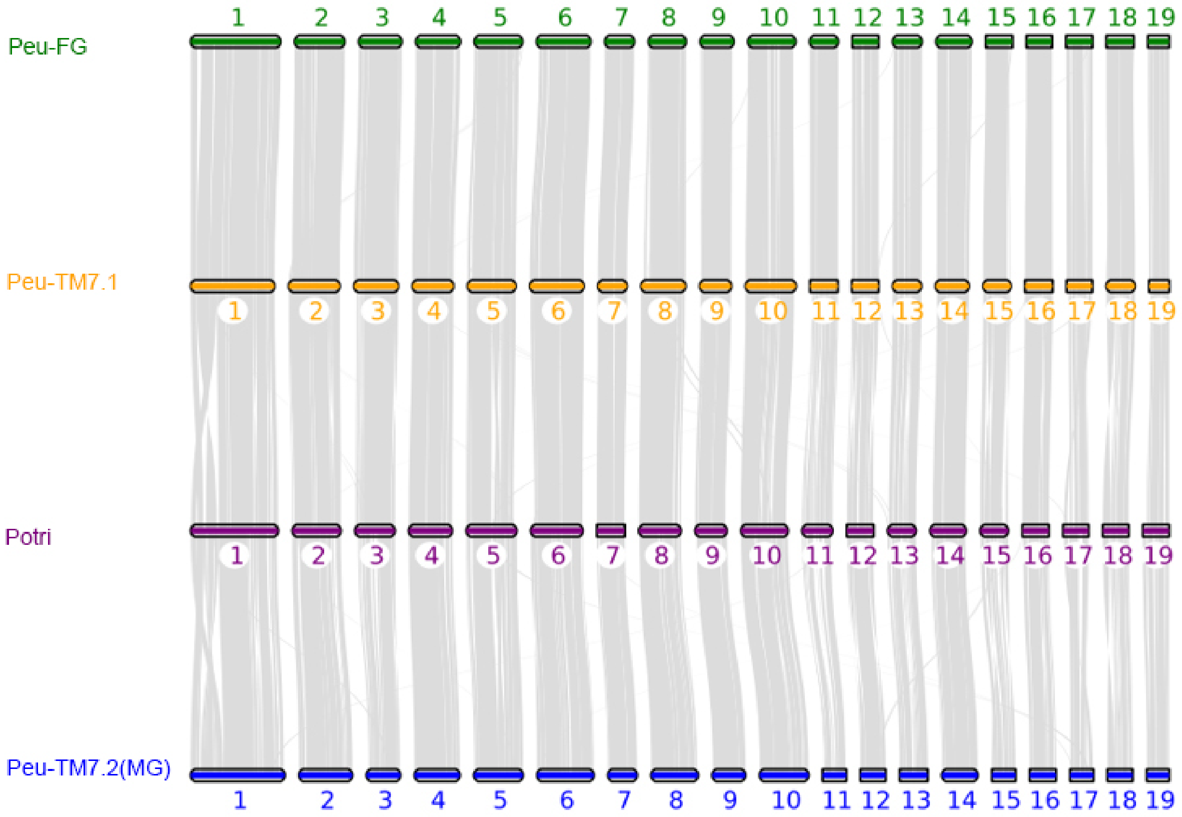

Supplementary Figure 2. Syntenic relationships among the female genome (Peu-FG), two male genomes (Peu-TM7.1 and Peu-TM7.2) of *P. euphratica*, and the genome of *P. trichocarpa* v3.1 (Potri).

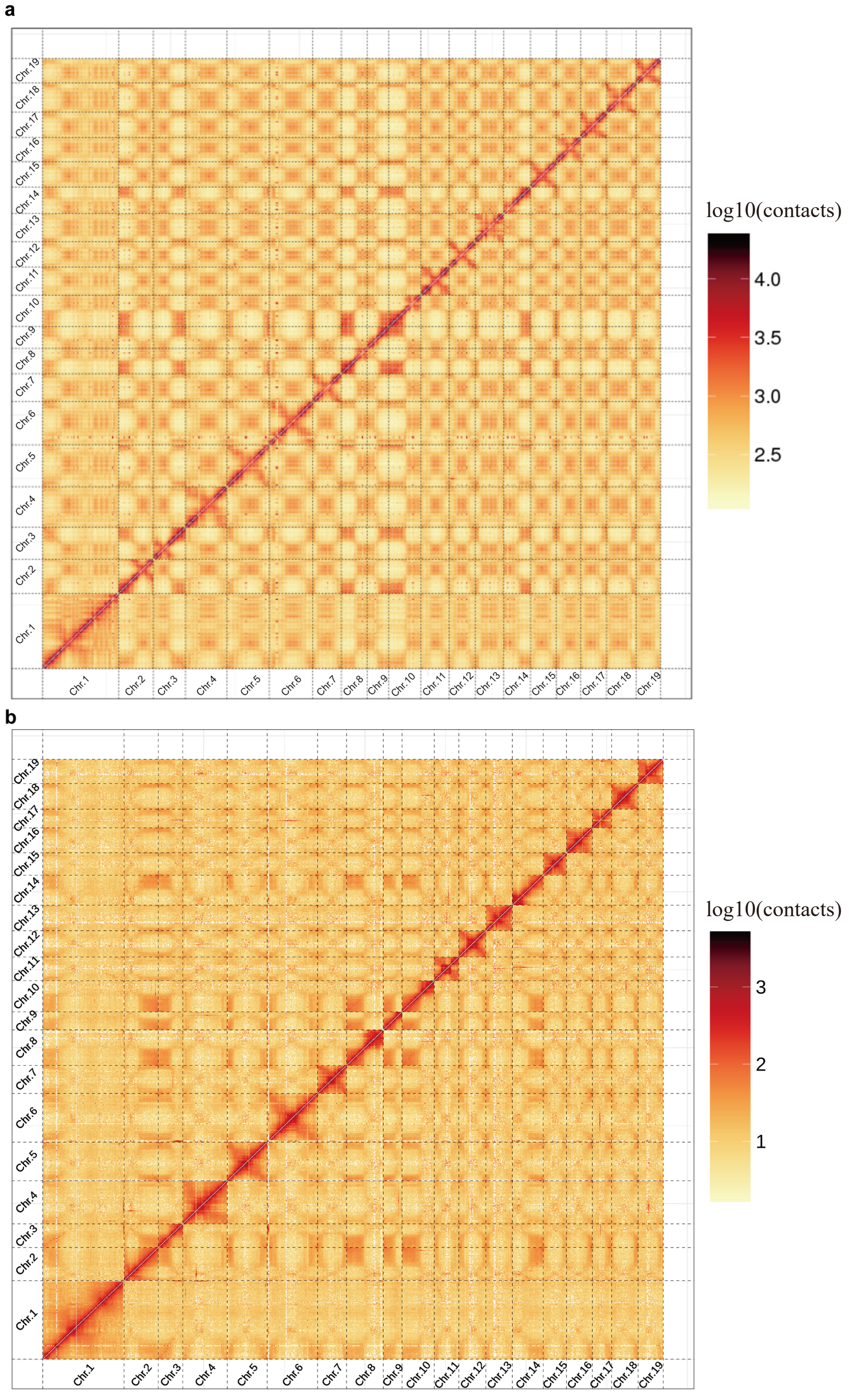

Supplementary Figure 3. Hi-C contact heat map. Hi-C map of the female genome (FG) (**a**) and the TM7.2 male genome (MG) (**b**) showing genome-wide all-by all interactions.

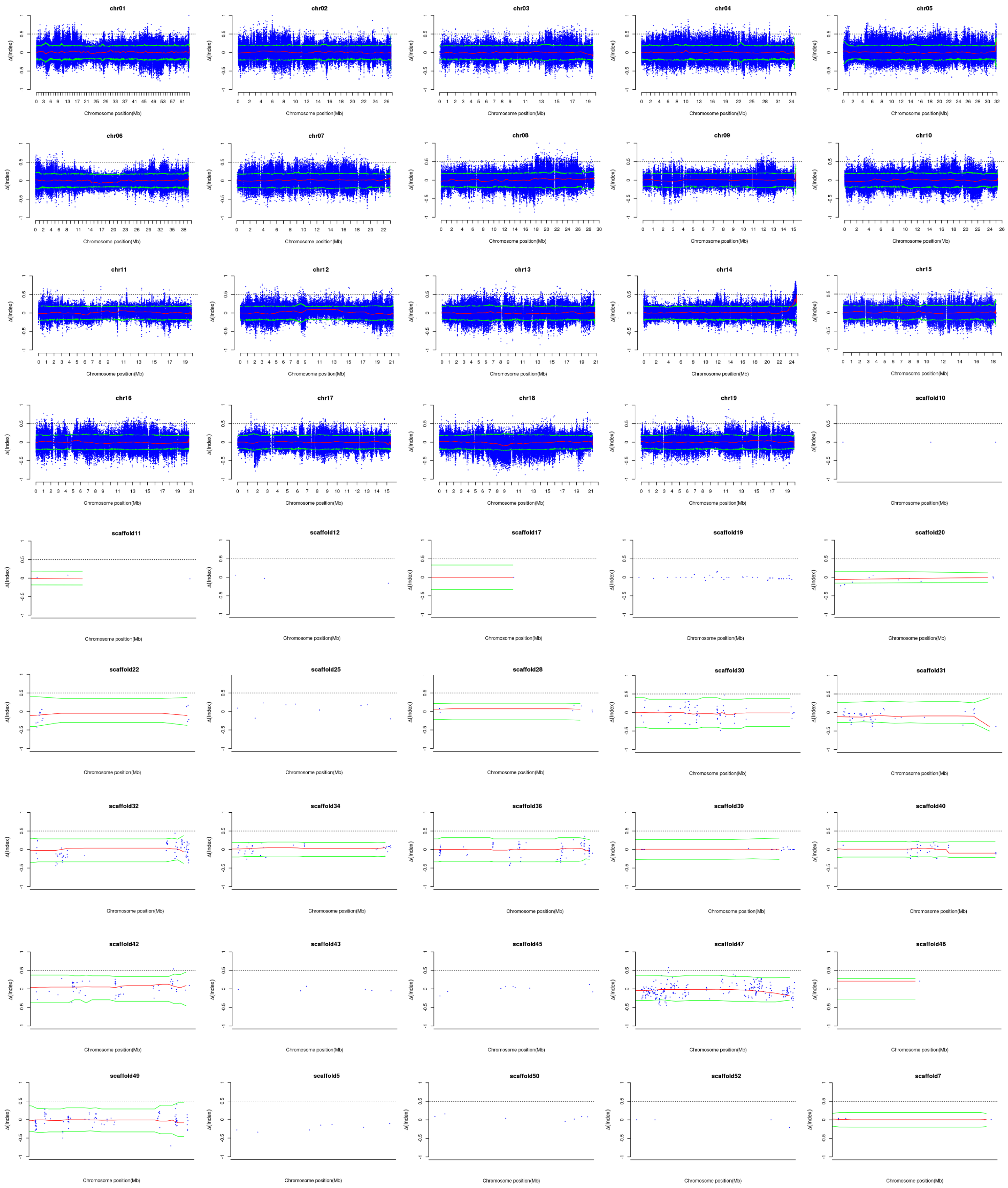

Supplementary Figure 4. ΔSNP_index calculated with reference to the MG. Curves in red are indexes calculated with 1 Mb sliding window and 1 kb step size. Green lines indicate the cutoff of 95% confidence interval.

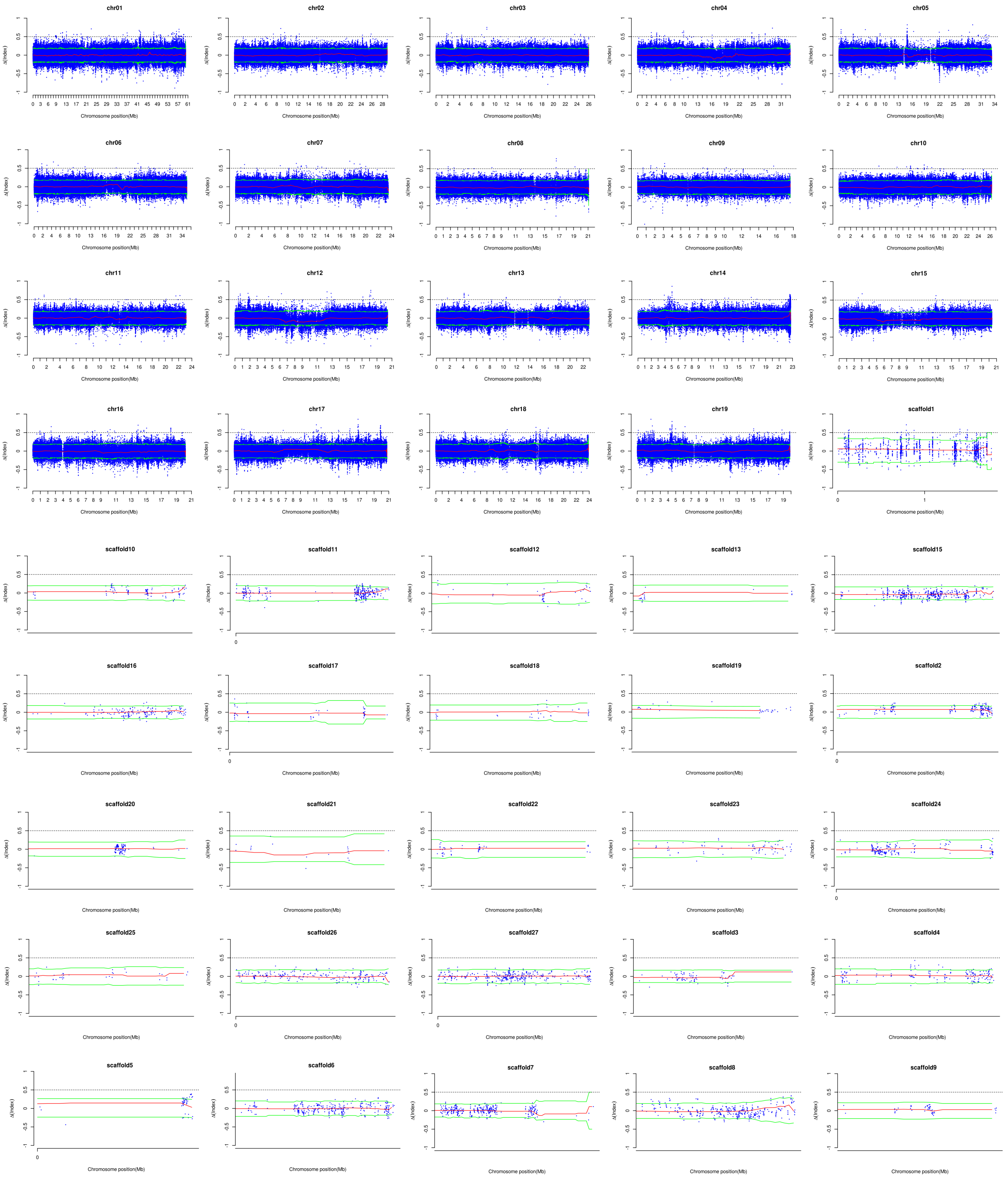

Supplementary Figure 5. ΔSNP_index calculated with reference to the FG. Curves in red are indexes calculated with 1 Mb sliding window and 1 kb step size. Green lines indicate the cutoff of 95% confidence interval.

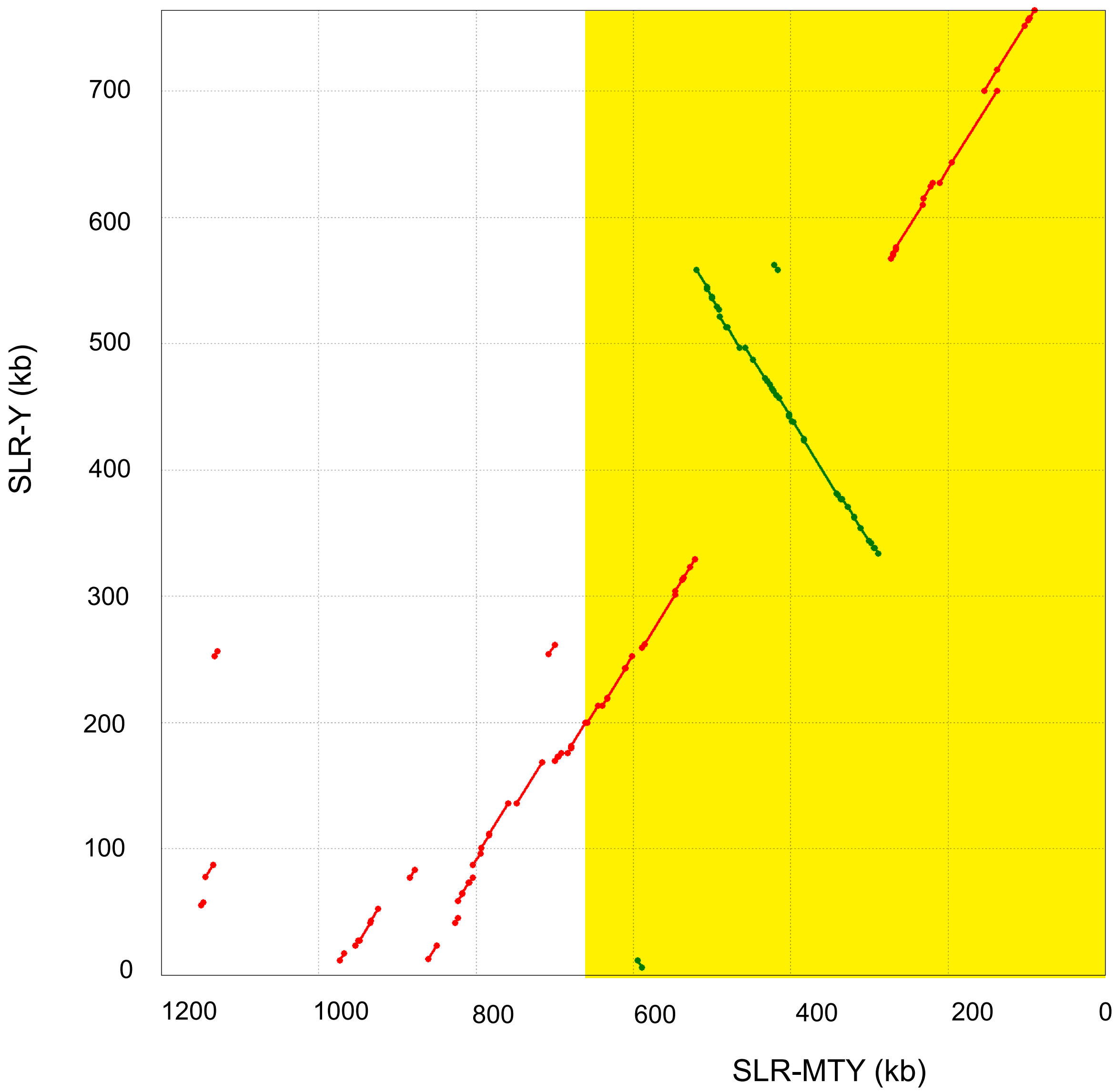

Supplementary Figure 6. Syntenic analysis of SLR-Y recognized in this study and SDR identified in Yang et al. [1] (denoted as “SLR-MTY” and indicated as yellow region) for *P. euphratica*. The red line represents colinear segments between SLR-MTY and SLR-Y in the same orientation; blue line represents colinear segments between SLR-MTY and SLR-Y in the inverted orientation.

1 Yang, W. *et al.* A general model to explain repeated turnovers of sex determination in the Salicaceae. *Molecular Biology and Evolution* **38**, 968-980, doi:10.1093/molbev/msaa261 (2020).

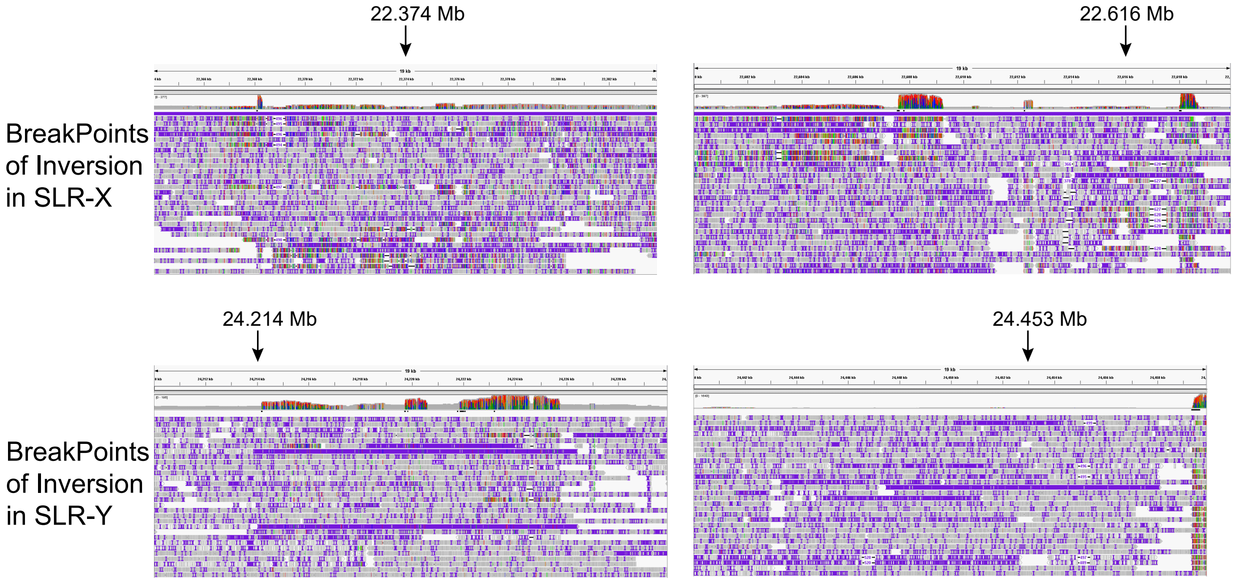

Supplementary Figure 7. PacBio long reads span over the breakpoints (arrows) of the inversion between SLR-X and SLR-Y.

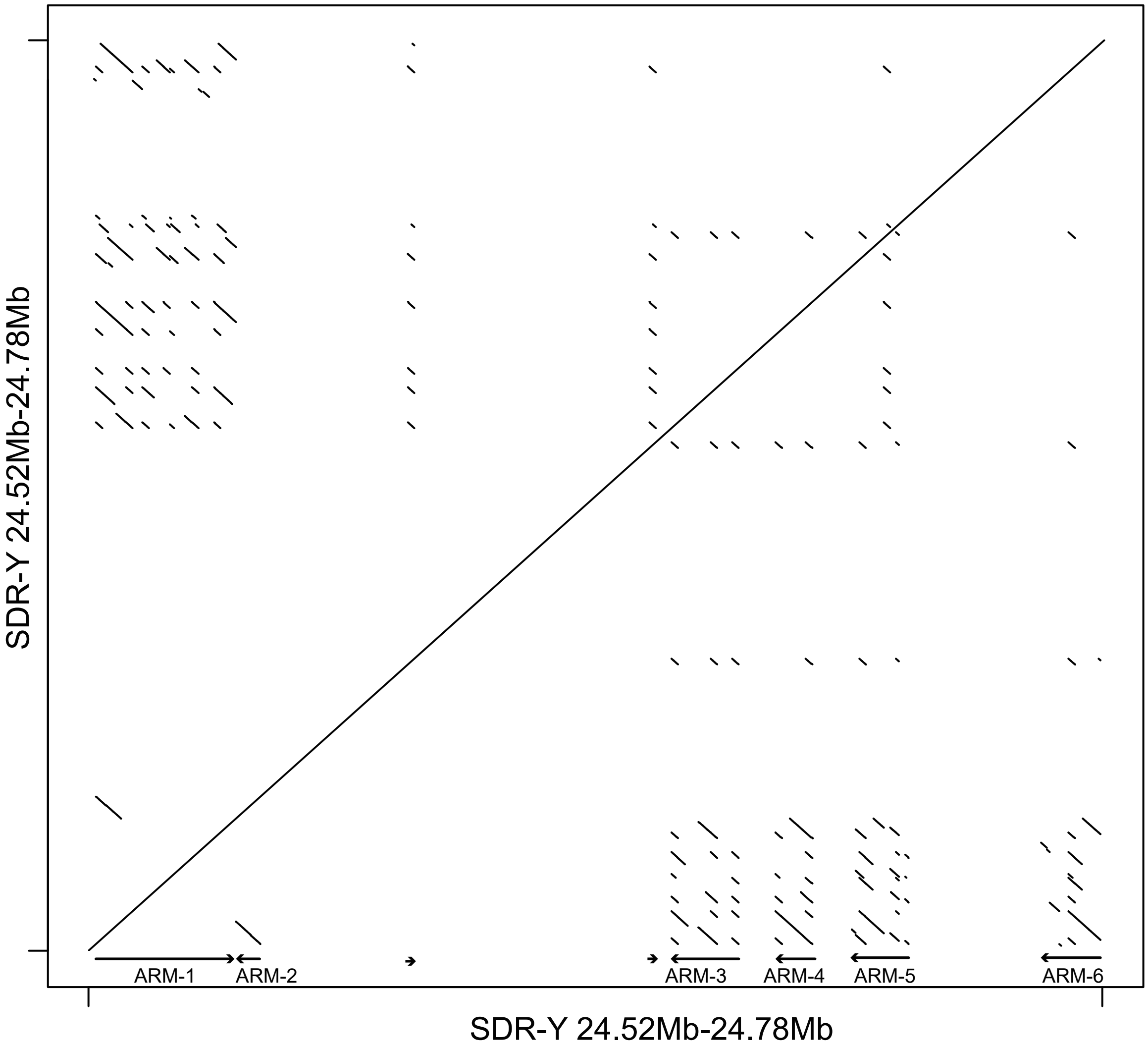

Supplementary Figure 8. Palindromic region for SLR-Y in *P. euphratica*.

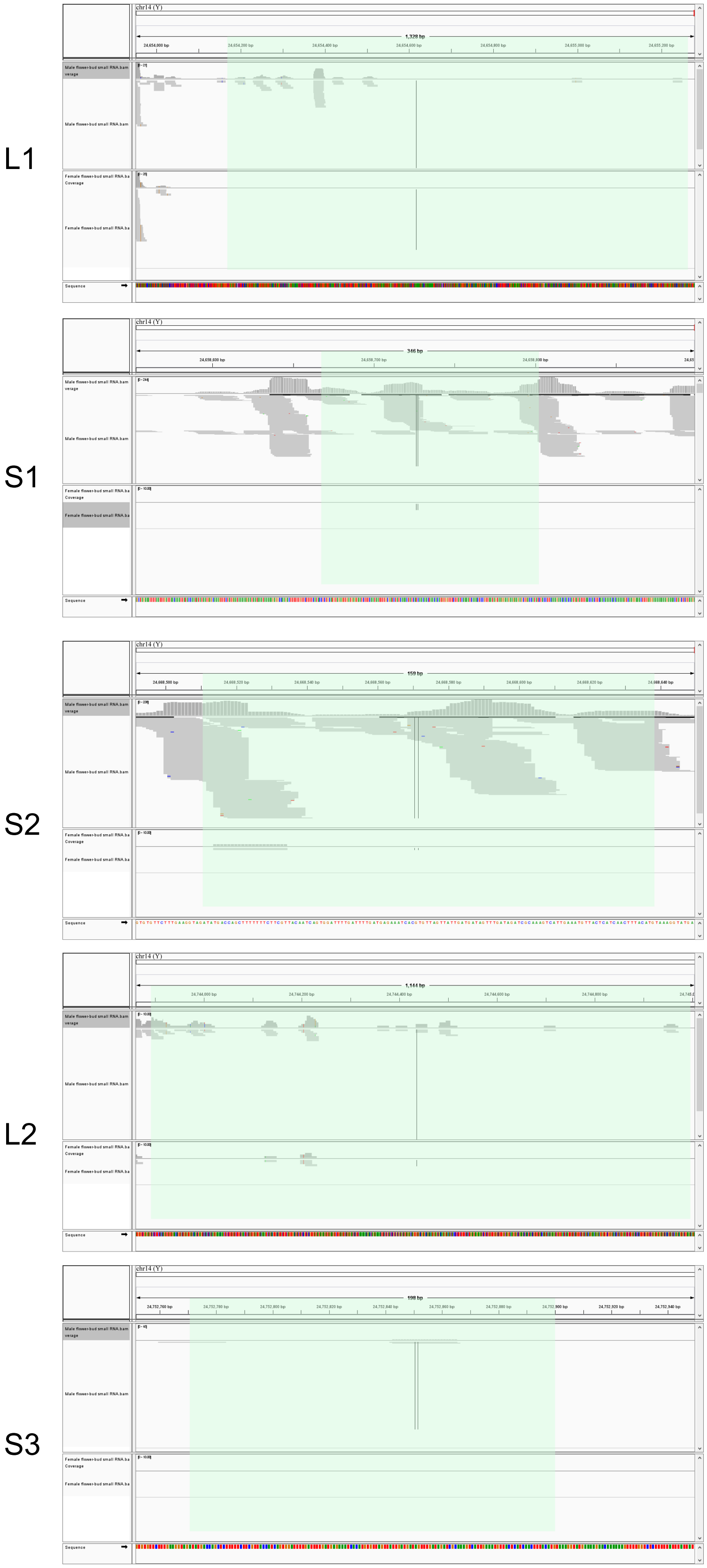

Supplementary Figure 9. Reads from small RNA-seq of female and male flower buds mapping to the two large segments L1–L2 and the small segments S1–S3 of *ARR17* in *P. euphratica*.

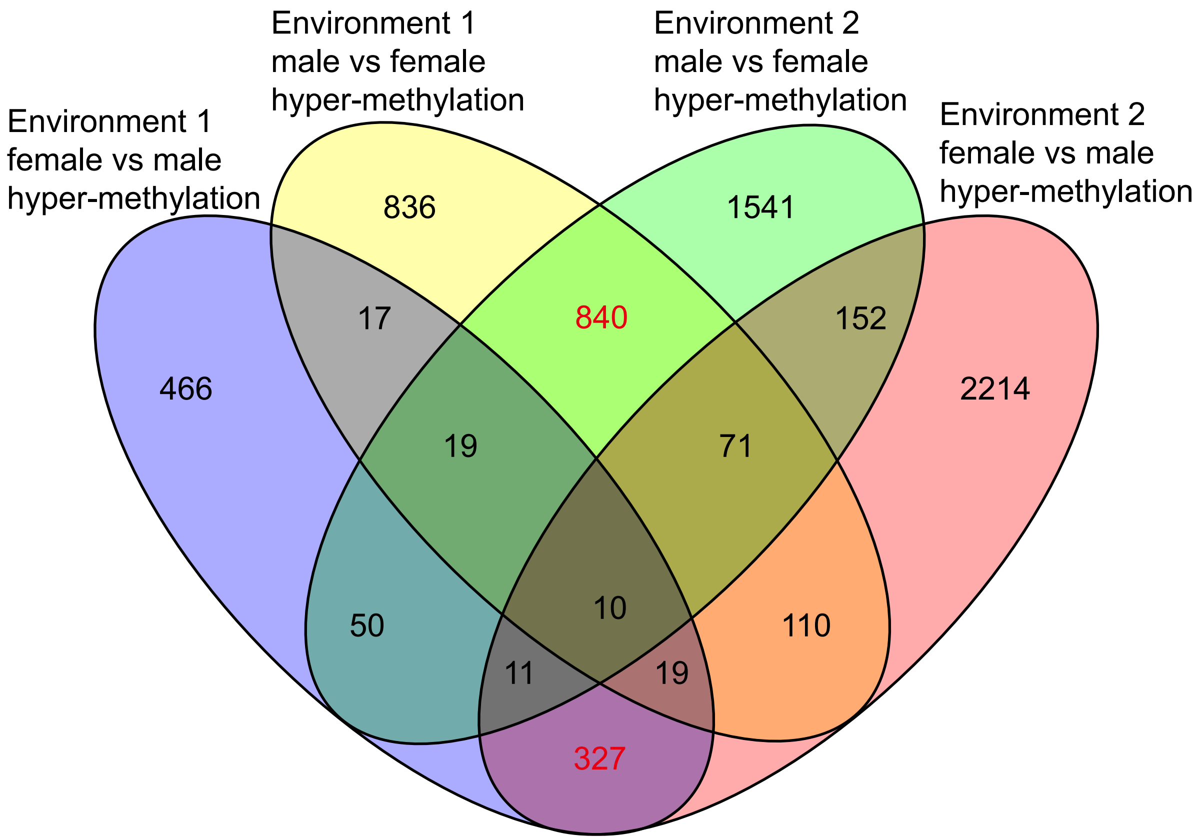

Supplementary Figure 10. Sex-specific differentially methylated genes in stem for *P. euphratica*. Red numbers indicate that the male/female hyper-methylated gene shared between environment 1 and environment 2.

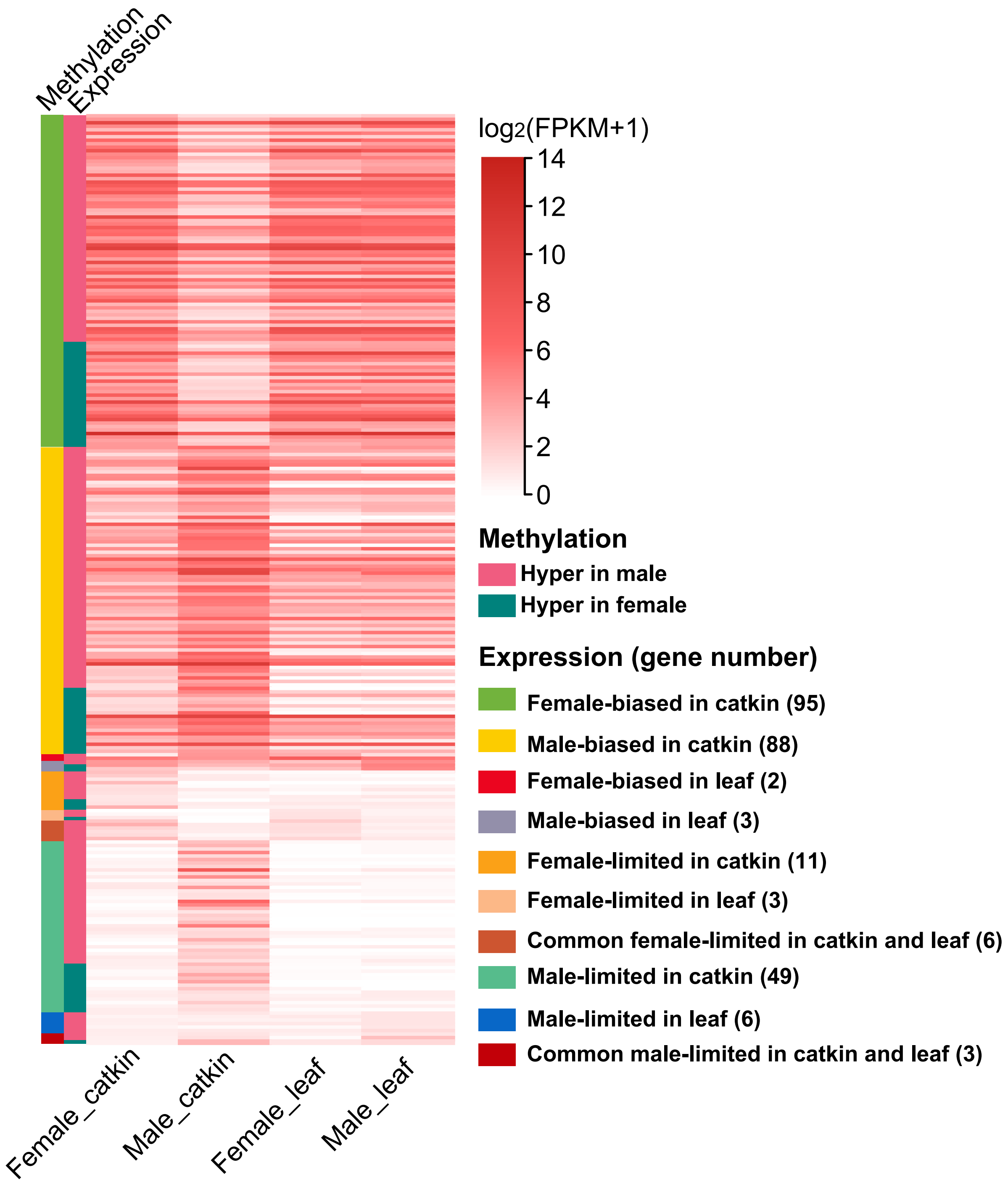

Supplementary Figure 11. Sexual dimorphism in expression of sex differentially methylated genes.
