# Supplementary material for: Chromosome-scale assemblies of the male and female Populus euphratica genomes reveal the molecular basis of sex determination and sexual dimorphism
Source: Commun Biol. 2022 Nov 4;5:1186. doi: 10.1038/s42003-022-04145-7 (PMC9636151; doi:10.1038/s42003-022-04145-7)
Supplement: Supplementary file 3 — Description of Additional Supplementary Data [file 42003_2022_4145_MOESM3_ESM.docx]

**Description of Additional Supplementary Files**

**File name:** Supplementary Data 1

**Description:** List of P. euphratica samples used in the study

**File name:** Supplementary Data 2

**Description:** Genome surveys of 10 female and 10 male P. euphratica individuals.

**File name:** Supplementary Data 3

**Description:** Assembly statistics for the newly assembled genomes in this study and the previously reported genome (Zhang et al. 2020) of P. euphratica.

**File name:** Supplementary Data 4

**Description:** Hi-C data quality report.

**File name:** Supplementary Data 5

**Description:** Chromosome and scaffold numbers of the assembled genomes of P. euphratica in this study and the previously reported genome (Zhang et al. 2020).

**File name:** Supplementary Data 6

**Description:** BUSCO assessment of the FG and MG in this study.

**File name:** Supplementary Data 7

**Description:** Classification of transposons in the FG and MG of P. euphratica

**File name:** Supplementary Data 8

**Description:** Annotation summary of predicted genes in the FG and MG of P. euphratica.

**File name:** Supplementary Data 9

**Description:** : ncRNA annotation in the FG and MG of P. euphratica.

**File name:** Supplementary Data 10

**Description:** data from female and male bulk DNA samples of P. euphratica.

**File name:** Supplementary Data 11

**Description:** Mapping results of BSA_Female and BSA_Male to the FG and MG.

**File name:** Supplementary Data 12

**Description:** Numbers of SNPs detected by BSA using FG and MG as the reference, respectively.

**File name:** Supplementary Data 13

**Description:** Location of candidate SLRs based on SNP-index in reference to the FG and MG.

**File name:** Supplementary Data 14

**Description:** Alignment between SLR-Y and its counterpart region on the X chromosome. Two positions of one chromosome in bold indicate the inversed region.

**File name:** Supplementary Data 15

**Description:** Annotation of the repeat elements in the specific regions on the sex chromosome.

**File name:** Supplementary Data 16

**Description:** Segments of ARR17 (PeuTM19G01068) on chromosome 14.

**File name:** Supplementary Data 17

**Description:** Supplementary Data 17. Difference of Methylation level on ARR17 between male and female (PeuTM19G01068).

**File name:** Supplementary Data 18

**Description:** Ka/Ks between exons of ARR17 (PeuTM19G01068) and segments of ARR17 in SLR-Y

**File name:** Supplementary Data 19

**Description:** Biological process enrichment of sex-specific methylated genes shared between two environments in young stems.

**File name:** Supplementary Data 20

**Description:** Homologous genes pairs in SLR-Y and SLR-X of P. euphratica. Genes in the inverted region are marked in bold.

**File name:** Supplementary Data 21

**Description:** Genes specific to SLR-Y of P. euphratica, compared to the equivalent region on the X chromosome. Genes in the inverted region are marked in bold.

**File name:** Supplementary Data 22

**Description:** Genes specific to SLR-X of P. euphratica, compared to SLR-Y. Genes in the inverted region are marked in bold.

**File name:** Supplementary Data 23

**Description:** Gene IDs, expression analysis and GO terms related to expression in the catkins and leaves of female and male P. euphratica individuals, and ortholog of each gene in P. trichocarpa V4 and its functional term.

**File name:** Supplementary Data 24

**Description:** Sex differentially expressed genes in catkins and leaves of P. euphratica. FALSE, not significantly different; UP, upregulated expression in that group; DOWN, downregulated gene (P-adj < 0.05, Up = Log2(Fold Change) > 1, Down = Log2(Fold Change) < –1).

**File name:** Supplementary Data 25

**Description:** Top 20 enriched biological processes of SLGs in catkins.

**File name:** Supplementary Data 26

**Description:** Significantly enriched biological processes for SLGs in leaves (P < 0.05).

**File name:** Supplementary Data 27

**Description:** Significantly enriched biological process for sex-limited gene shared between catkin and leaf (P < 0.05).

**File name:** Supplementary Data 28

**Description:** Top 20 enriched biological processes of SBGs in catkins.

**File name:** Supplementary Data 29

**Description:** Enrichment in biological processes of SBGs in leaves.

**File name:** Supplementary Data 30

**Description:** Common SBGs in leaves and catkins of P. euphratica.

**File name:** Supplementary Data 31

**Description:** Summary statistics of RNA-seq reads mapping to the P. euphratica FG.
