# Supplementary material for: Chromosome-scale assemblies of the male and female Populus euphratica genomes reveal the molecular basis of sex determination and sexual dimorphism
Source: Commun Biol. 2022 Nov 4;5:1186. doi: 10.1038/s42003-022-04145-7 (PMC9636151; doi:10.1038/s42003-022-04145-7)
Supplement: Supplementary file 5 — Reporting Summary-New [file 42003_2022_4145_MOESM5_ESM.pdf]

nature portfolio

Corresponding author(s): Zhijun Li, Peipei Jiao

Last updated by author(s): Oct 5, 2022

Reporting Summary

Nature Portfolio wishes to improve the reproducibility of the work that we publish. This form provides structure for consistency and transparency in reporting. For further information on Nature Portfolio policies, see our Editorial Policies and the Editorial Policy Checklist.

Statistics

For all statistical analyses, confirm that the following items are present in the figure legend, table legend, main text, or Methods section.

n/a	Confirmed	
☐	☒	The exact sample size (*n*) for each experimental group/condition, given as a discrete number and unit of measurement
☐	☒	A statement on whether measurements were taken from distinct samples or whether the same sample was measured repeatedly
☐	☒	The statistical test(s) used AND whether they are one- or two-sided<br>*Only common tests should be described solely by name; describe more complex techniques in the Methods section.*
☒	☐	A description of all covariates tested
☐	☒	A description of any assumptions or corrections, such as tests of normality and adjustment for multiple comparisons
☐	☒	A full description of the statistical parameters including central tendency (e.g. means) or other basic estimates (e.g. regression coefficient) AND variation (e.g. standard deviation) or associated estimates of uncertainty (e.g. confidence intervals)
☐	☒	For null hypothesis testing, the test statistic (e.g. *F*, *t*, *r*) with confidence intervals, effect sizes, degrees of freedom and *P* value noted<br>*Give P values as exact values whenever suitable.*
☒	☐	For Bayesian analysis, information on the choice of priors and Markov chain Monte Carlo settings
☒	☐	For hierarchical and complex designs, identification of the appropriate level for tests and full reporting of outcomes
☒	☐	Estimates of effect sizes (e.g. Cohen's *d*, Pearson's *r*), indicating how they were calculated

Our web collection on statistics for biologists contains articles on many of the points above.

Software and code

Policy information about availability of computer code

Data collection

No commercial/custom code was used for data collection.

Data analysis

Genome assembly and evaluation.
De novo assembly of the long reads generated from the PacBio SMRT Sequencing was performed using FALCON v1.0. Before assembly, errors in the PacBio reads were corrected with the FALCON pipeline. The corrected reads were then aligned to each other and assembled into genomic contigs using FALCON with the following parameters: length_cutoff_pr = 5000, max_diff = 65, max_cov = 75. The produced primary contigs (p-contigs) were then polished using Quiver by incorporating all SMRT reads. Then, Pilon was used to perform a second round of error correction with short paired-end reads generated from Illumina Nova seq 6000 sequencing. Subsequently, the Purge Haplotigs pipeline was used to remove redundant sequences resulting from heterozygosity in the female individual. Because the male genome had a relatively high degree of heterozygosity, most parts of the genome were assembled as separate primary contigs (haploid assembly), which produced contigs twice the size of the haploid genome (1.03 Gb), rather than a single haplotype-fused contig. Next, the draft diploid assembly and the alignment file of PacBio long-reads mapped to the assembly were loaded into the Purge haplotigs pipeline, resulting in curated primary contigs and reassigned haplotigs.
Hi-C reads were used to improve the quality of assemblies. To avoid reads with artificial bias, we removed the following: (a) reads with ≥10% unidentified nucleotides (N); (b) reads with >10 nt aligned to the adapter, allowing ≤10% mismatches; and (c) reads with >50% bases having phred quality <5. The filtered Hi-C reads were aligned against the contig assemblies with BWA version 0.7.8. Reads were excluded from subsequent analysis if they did not align within 500 bp of a restriction site or did not uniquely map, and the number of Hi-C read pairs linking each pair of scaffolds was tabulated. LACHESIS (http://shendurelab.github.io/LACHESIS/) was used to perform hierarchical agglomerative clustering and assign scaffolds to 19 groups. Juicebox v1.9.8 was finally used to order the scaffolds in each group.
BUSCO was used to evaluate the completeness of the assembly and of the annotation by mapping the genome sequence and annotated protein sequences to the database embryophyta_odb10.

Genome annotation.

Repeat sequences in the genomes were annotated using both ab initio and homology-based search methods. For ab initio predictions, RepeatModeler (http://www.repeatmasker.org/RepeatModeler/), RepeatScout (http://www.repeat masker.org/), and LTR_FINDER (http://tlife.fudan.edu.cn/ltr_finder/) were used to discover TEs and to build a TE library. The resulting TE library and a known repeat library (Repbase V15.02, homolog-based) were then subjected to RepeatMasker (http://www.repeatmasker.org/) for repeat identification. For homology-based predictions, RepeatProteinMask (http://www.repeatmasker.org/) was employed to detect TEs in the genome by comparisons against the TE protein database. A consensus list of TEs was generated from the combination of the two methods. Tandem repeats were identified in the genome using Tandem Repeats Finder (version 4.07b). All repetitive regions except tandem repeats were soft-masked for gene prediction.
Based on the repeat-masked genomes, protein-coding genes were annotated with a combination of RNA-seq mapping, homology-based gene prediction, and ab initio prediction. For RNA-seq prediction, the Illumina RNA-seq data from stems, catkins, leaves and petioles of the female and male individuals were aligned to the assembled female and male genome using Tophat version 2.0.13 to identify exons region and splice positions, respectively. The alignment results were then used as input for Cufflinks version 2.1.1 to assemble transcripts to the gene models. In addition, RNA-seq data were assembled by Trinity version 2.1.1, creating several pseudo-ESTs. These pseudo-ESTs were also mapped to the assembled genome by BLAT and gene models were predicted using PASA. For protein homolog search, the protein sequences of nine homologous species (Populus euphratica, Populus trichocarpa, Populus pruinosa, Actinidia chinensis, Carica papaya, Juglans regia, Jatropha curcas, Arabidopsis thaliana, Oryza sativa) were aligned against to the genome using TBLASTN (E-value 1E-05). Genewise version 2.2.0 was employed to predict gene models based on these alignments. Ab initio prediction was performed to predict gene structure using five gene prediction programs including Augustus v3.0.2, Genescan v1.0, Geneid v1.4, GlimmerHMM v3.0.2 and SNAP version 2013-02-16. A weighted and non-redundant gene set was generated by EVidenceModeler (EVM, version 1.1.1), which merged all genes models predicted by the above three approaches. Finally, PASA was used to adjust the gene models generated by EVM.
The functional assignments of the P. euphratica genes were conducted by Basic Local Alignment Search Tool (BLAST) against public protein databases, including SwissProt (https://web.expasy.org/docs/swiss-prot_guideline.html), GO, NR, InterPro, Pfam and KEGG (https://www.kegg.jp/). ncRNAs were predicted using de novo and homology search methods.

Identification of the SLR.
DNA sequencing libraries were prepared from bulk genomic DNA from 100 P. euphratica female individuals and 96 P. euphratica male individuals collected in the wild and sequenced, generating 134-fold and 119-fold coverage, respectively, on an Illumina Nova Seq 6000 instrument. The clean bulked reads (BSA-M and BSA-F) were aligned and mapped onto the de novo assembled genome from this study by Burrows-Wheeler Aligner software (BWA). Alignment files were converted to BAM files using SAMtools software with parameter setting as: -bS -t. SNP calling and filtering were performed using the Unified Genotype function and the Variant Filtration in GATK software. ANNOVAR software was used to annotate SNPs based on the annotation file for the de novo assembled genome.
To identify the SLR, the ratio of different reads was calculated between the two bulked pools to obtain the SNP-index of each nucleotide position. Subsequently, the difference in SNP-indexes between the two pools was calculated as ΔSNP-index. A sliding window method was used to present the ΔSNP-indexes across the whole genome, with a window size of 1 Mb and a step size of 10 kb. One thousand permutation tests were performed, and the 95% confidence level was selected as the screening threshold.
Moreover, palindromic repeats in SLR-Y were detected based on self-alignment using LASTZ 1.03.66 with the following options: --gapped --exact = 100 --step = 20. Additionally, small RNA data of female flower buds (BIG Bio Sample ID: SAMC206760) and male flower buds (BIG Bio Sample ID: SAMC206761) of P. euphratica, downloaded from BIG, were mapped to the SLR-Y and the counterpart region on X (SLR-X) with Bowtie v1.2.2. The mapping results were visualized in the Integrative Genomic Viewer tool. Homologous gene pairs between SLR-Y and SLR-X were identified by performing a reciprocal BLASTP with default parameters.

Transcriptome sequencing and identification of SLGs/SBGs.
Both leaf and catkin samples from five male and five female individuals of Populus euphratica were collected from natural populations in Tarim Basin, Xinjiang province, China, in early spring 2019. Whole male and female catkins were collected when the catkin was fully expanded but had not flowered yet. Young leaves were sampled when they were fully expanded. The tissue samples were frozen in liquid nitrogen and stored at -80°C until RNA extraction.
Total RNA was extracted from each sample using the RNAprep Pure Plant Kit (Tiangen), and genomic DNA contamination was removed using RNase-Free DNase I (Tiangen). Isolated purified RNA was used for cDNA library construction, with fragment lengths of approximately 250 bp, using the NEBNext Ultra RNA Library Prep Kit for Illumina (New England Biolabs, Ipswich, MA, USA), according to the manufacturer's instructions.
Sequencing was performed on an Illumina Nova Seq 6000 instrument and 150 bp paired-end reads were generated. Raw reads were trimmed by removing adapter sequences, reads with more than 5% of unknown base calls (N) and low-quality bases (base quality less than 5, $Q \leq 5$). Clean paired-end reads were aligned to the de novo assembled genomes from this study using HISAT2 v2.0.4 with default parameters (Supplementary Table S21). HTSeq v0.6.1 was used to count the read numbers mapped to each gene.
Gene expression was quantified using fragments per kilobase of transcript sequence per millions base pairs (FPKM). Expressed genes in one tissue were filtered for FPKM >1. In one tissue, genes were classified as exhibiting a sex-limited expression profile if they were expressed in one sex only. Genes with sex-biased expression were expressed in both sexes but differentially expressed in one or the other sex. Normalized read counts for all detected genes were used to identify differentially expressed genes by the negative binomial distribution model implemented in DESeq software v1.10.1. Differentially expressed genes were identified with a |log2 (fold change)| > 1 and adjusted P value <0.05 using the Benjamini and Hochberg correction.
Furthermore, GO enrichment analysis was performed with the GOseq R package v1.24.0 with correction for gene length bias. Enrichment P values were estimated with Fisher's Exact Test.

Whole-genome bisulfite sequencing and differential sex methylation analysis.
P. euphratica samples used for methylation analysis were collected from two environmentally divergent fields and consisted of three males and three females in each environment. Xylem was chosen because it is considered a consistent and homogeneous tissue with little phenotypic plasticity relative to the environment, unlike leaves. Genomic DNA was extracted and fragmented by sonication to 200-300 bp with Covaris S220, followed by end repair and adenylation. Cytosine-methylated barcodes were ligated to sonicated DNA following the manufacturer's instructions. These ligated fragments were treated twice with bisulfite using EZ DNA Methylation-Gold Kit (Zymo Research), before the resulting single-stranded DNA fragments were PCR amplified using KAPA HiFi HotStart Uracil + ReadyMix (2X). The prepared library was sequenced giving average depth of 30[illegible] on an Illumina Nova seq 6000 instrument.
The raw sequencing reads were trimmed through Trimmomatic v0.36 using the parameters: SLIDINGWINDOW: 4:15; LEADING:3, TRAILING:3; ILLUMINACLIP: adapter.fa: 2: 30: 10 ; MINLEN:36. The trimmed reads for each individual were mapped to corresponding corrected pseudo-reference genome, which is generated by replacing the FG assembled in this study with filtered SNP variants, using bowtie2, and duplicated reads were subsequently removed from the BAM files using SAMtools software with the command: rmdup. Finally, methylation levels were called and extracted using Bismark v0.16.3. Differentially methylated regions (DMRs) between female and male individuals in each environment were identified using the DSS package. According to the distribution of DMRs across the genome, genes were defined as being

related to DMRs when the gene body region (from translation start site to translation termination site) or promoter region (2 kb upstream from the translation start site) overlapped with the DMRs. The genes related to sex-specific DMRs and shared between two environments were used for GO enrichment analysis.

Statistics and reproducibility.
One thousand permutation test with the screening threshold of 95% confidence level was performed to identified the sex-linked region in whole genome. Differentially expressed genes and differentially methylated level were taken from distinct biological replicates. $P < 0.05$ after adjustment using the Benjamini and Hochberg correction is considered statistically significant. GO enrichment analysis was performed with the GOseq R package v1.24.0 with correction for gene length bias. Fisher's Exact Test was performed on Enrichment and $P < 0.05$ is considered statically significant.

For manuscripts utilizing custom algorithms or software that are central to the research but not yet described in published literature, software must be made available to editors and reviewers. We strongly encourage code deposition in a community repository (e.g. GitHub). See the Nature Portfolio guidelines for submitting code & software for further information.

Data

Policy information about availability of data

All manuscripts must include a data availability statement. This statement should provide the following information, where applicable:

- Accession codes, unique identifiers, or web links for publicly available datasets
- A description of any restrictions on data availability
- For clinical datasets or third party data, please ensure that the statement adheres to our policy

The genome sequencing raw data, BSA sequencing data, RNA-seq data, bisulfite sequencing data and genome assemblies and annotations for female and male P. euphratica in this study have been deposited in the Genome Warehouse in National Genomics Data Center, Beijing Institute of Genomics, Chinese Academy of Sciences / China National Center for Bioinformation, under BioProject ID PRJCA006811 that is publicly accessible at http://bigd.big.ac.cn/bioproject.

Field-specific reporting

Please select the one below that is the best fit for your research. If you are not sure, read the appropriate sections before making your selection.

☒ Life sciences ☐ Behavioural & social sciences ☐ Ecological, evolutionary & environmental sciences

For a reference copy of the document with all sections, see nature.com/documents/nr-reporting-summary-flat.pdf

Life sciences study design

All studies must disclose on these points even when the disclosure is negative.

Sample size

Leaf sample of the female P. euphratica TF1-136, and Leaf sample of the male P. euphratica TM7 in Tarim Basin were used for De novo genome sequencing. The leaf, petiole, catkin and stem on their own individual was used for RNA-seq, which generated data was used to help the annotation of thier genome.

For bulked segregant analysis to identify the sex-linked region, 100 female trees and 96 male trees were collected in the wild.

For the sex dimorphism analysis in transcriptomic level, Leaves and catkins of five female P. euphartica and five male P. euphratica were used for RNA-seq.

For the sex dimorphism analysis in methylation level, stems of each three female and male trees in the field 1 and field 2 was used for Whole-genome bisulfite sequencing.

Data exclusions

No data were excluded from the analysis.

Replication

Biological replicates were used as required and described in the methods section.

Randomization

Not applicable.

Blinding

Not applicable.

Behavioural & social sciences study design

All studies must disclose on these points even when the disclosure is negative.

Study description

Briefly describe the study type including whether data are quantitative, qualitative, or mixed-methods (e.g. qualitative cross-sectional, quantitative experimental, mixed-methods case study).

Research sample | *State the research sample (e.g. Harvard university undergraduates, villagers in rural India) and provide relevant demographic information (e.g. age, sex) and indicate whether the sample is representative. Provide a rationale for the study sample chosen. For studies involving existing datasets, please describe the dataset and source.*

Sampling strategy | *Describe the sampling procedure (e.g. random, snowball, stratified, convenience). Describe the statistical methods that were used to predetermine sample size OR if no sample-size calculation was performed, describe how sample sizes were chosen and provide a rationale for why these sample sizes are sufficient. For qualitative data, please indicate whether data saturation was considered, and what criteria were used to decide that no further sampling was needed.*

Data collection | *Provide details about the data collection procedure, including the instruments or devices used to record the data (e.g. pen and paper, computer, eye tracker, video or audio equipment) whether anyone was present besides the participant(s) and the researcher, and whether the researcher was blind to experimental condition and/or the study hypothesis during data collection.*

Timing | *Indicate the start and stop dates of data collection. If there is a gap between collection periods, state the dates for each sample cohort.*

Data exclusions | *If no data were excluded from the analyses, state so OR if data were excluded, provide the exact number of exclusions and the rationale behind them, indicating whether exclusion criteria were pre-established.*

Non-participation | *State how many participants dropped out/declined participation and the reason(s) given OR provide response rate OR state that no participants dropped out/declined participation.*

Randomization | *If participants were not allocated into experimental groups, state so OR describe how participants were allocated to groups, and if allocation was not random, describe how covariates were controlled.*

Ecological, evolutionary & environmental sciences study design

All studies must disclose on these points even when the disclosure is negative.

Study description | *Briefly describe the study. For quantitative data include treatment factors and interactions, design structure (e.g. factorial, nested, hierarchical), nature and number of experimental units and replicates.*

Research sample | *Describe the research sample (e.g. a group of tagged Passer domesticus, all Stenocereus thurberi within Organ Pipe Cactus National Monument), and provide a rationale for the sample choice. When relevant, describe the organism taxa, source, sex, age range and any manipulations. State what population the sample is meant to represent when applicable. For studies involving existing datasets, describe the data and its source.*

Sampling strategy | *Note the sampling procedure. Describe the statistical methods that were used to predetermine sample size OR if no sample-size calculation was performed, describe how sample sizes were chosen and provide a rationale for why these sample sizes are sufficient.*

Data collection | *Describe the data collection procedure, including who recorded the data and how.*

Timing and spatial scale | *Indicate the start and stop dates of data collection, noting the frequency and periodicity of sampling and providing a rationale for these choices. If there is a gap between collection periods, state the dates for each sample cohort. Specify the spatial scale from which the data are taken*

Data exclusions | *If no data were excluded from the analyses, state so OR if data were excluded, describe the exclusions and the rationale behind them, indicating whether exclusion criteria were pre-established.*

Reproducibility | *Describe the measures taken to verify the reproducibility of experimental findings. For each experiment, note whether any attempts to repeat the experiment failed OR state that all attempts to repeat the experiment were successful.*

Randomization | *Describe how samples/organisms/participants were allocated into groups. If allocation was not random, describe how covariates were controlled. If this is not relevant to your study, explain why.*

Blinding | *Describe the extent of blinding used during data acquisition and analysis. If blinding was not possible, describe why OR explain why blinding was not relevant to your study.*

Did the study involve field work? ☐ Yes ☐ No

Field work, collection and transport

Field conditions | *Describe the study conditions for field work, providing relevant parameters (e.g. temperature, rainfall).*

Location | *State the location of the sampling or experiment, providing relevant parameters (e.g. latitude and longitude, elevation, water depth).*

Access & import/export | *Describe the efforts you have made to access habitats and to collect and import/export your samples in a responsible manner and in compliance with local, national and international laws, noting any permits that were obtained (give the name of the issuing authority, the date of issue, and any identifying information).*

Disturbance | *Describe any disturbance caused by the study and how it was minimized.*

Reporting for specific materials, systems and methods

We require information from authors about some types of materials, experimental systems and methods used in many studies. Here, indicate whether each material, system or method listed is relevant to your study. If you are not sure if a list item applies to your research, read the appropriate section before selecting a response.

Materials & experimental systems

n/a	Involved in the study
☒	☐ Antibodies
☒	☐ Eukaryotic cell lines
☒	☐ Palaeontology and archaeology
☒	☐ Animals and other organisms
☒	☐ Human research participants
☒	☐ Clinical data
☒	☐ Dual use research of concern

Methods

n/a	Involved in the study
☒	☐ ChIP-seq
☒	☐ Flow cytometry
☒	☐ MRI-based neuroimaging

Antibodies

Antibodies used

Describe all antibodies used in the study; as applicable, provide supplier name, catalog number, clone name, and lot number.

Validation

Describe the validation of each primary antibody for the species and application, noting any validation statements on the manufacturer's website, relevant citations, antibody profiles in online databases, or data provided in the manuscript.

Eukaryotic cell lines

Policy information about cell lines

Cell line source(s)

State the source of each cell line used.

Authentication

Describe the authentication procedures for each cell line used OR declare that none of the cell lines used were authenticated.

Mycoplasma contamination

Confirm that all cell lines tested negative for mycoplasma contamination OR describe the results of the testing for mycoplasma contamination OR declare that the cell lines were not tested for mycoplasma contamination.

Commonly misidentified lines
(See ICLAC register)

Name any commonly misidentified cell lines used in the study and provide a rationale for their use.

Palaeontology and Archaeology

Specimen provenance

Provide provenance information for specimens and describe permits that were obtained for the work (including the name of the issuing authority, the date of issue, and any identifying information). Permits should encompass collection and, where applicable, export.

Specimen deposition

Indicate where the specimens have been deposited to permit free access by other researchers.

Dating methods

If new dates are provided, describe how they were obtained (e.g. collection, storage, sample pretreatment and measurement), where they were obtained (i.e. lab name), the calibration program and the protocol for quality assurance OR state that no new dates are provided.

☐ Tick this box to confirm that the raw and calibrated dates are available in the paper or in Supplementary Information.

Ethics oversight

Identify the organization(s) that approved or provided guidance on the study protocol, OR state that no ethical approval or guidance was required and explain why not.

Note that full information on the approval of the study protocol must also be provided in the manuscript.

Animals and other organisms

Policy information about studies involving animals; ARRIVE guidelines recommended for reporting animal research

Laboratory animals

For laboratory animals, report species, strain, sex and age OR state that the study did not involve laboratory animals.

Wild animals

Provide details on animals observed in or captured in the field; report species, sex and age where possible. Describe how animals were caught and transported and what happened to captive animals after the study (if killed, explain why and describe method; if released, say where and when) OR state that the study did not involve wild animals.

Field-collected samples	*For laboratory work with field-collected samples, describe all relevant parameters such as housing, maintenance, temperature, photoperiod and end-of-experiment protocol OR state that the study did not involve samples collected from the field.*
Ethics oversight	*Identify the organization(s) that approved or provided guidance on the study protocol, OR state that no ethical approval or guidance was required and explain why not.*

Note that full information on the approval of the study protocol must also be provided in the manuscript.

Human research participants

Policy information about studies involving human research participants

Population characteristics	*Describe the covariate-relevant population characteristics of the human research participants (e.g. age, gender, genotypic information, past and current diagnosis and treatment categories). If you filled out the behavioural & social sciences study design questions and have nothing to add here, write "See above."*
Recruitment	*Describe how participants were recruited. Outline any potential self-selection bias or other biases that may be present and how these are likely to impact results.*
Ethics oversight	*Identify the organization(s) that approved the study protocol.*

Note that full information on the approval of the study protocol must also be provided in the manuscript.

Clinical data

Policy information about clinical studies

All manuscripts should comply with the ICMJE guidelines for publication of clinical research and a completed CONSORT checklist must be included with all submissions.

Clinical trial registration	*Provide the trial registration number from ClinicalTrials.gov or an equivalent agency.*
Study protocol	*Note where the full trial protocol can be accessed OR if not available, explain why.*
Data collection	*Describe the settings and locales of data collection, noting the time periods of recruitment and data collection.*
Outcomes	*Describe how you pre-defined primary and secondary outcome measures and how you assessed these measures.*

Dual use research of concern

Policy information about dual use research of concern

Hazards

Could the accidental, deliberate or reckless misuse of agents or technologies generated in the work, or the application of information presented in the manuscript, pose a threat to:

No	Yes	
☐	☐	Public health
☐	☐	National security
☐	☐	Crops and/or livestock
☐	☐	Ecosystems
☐	☐	Any other significant area

Experiments of concern

Does the work involve any of these experiments of concern:

No	Yes	
☐	☐	Demonstrate how to render a vaccine ineffective
☐	☐	Confer resistance to therapeutically useful antibiotics or antiviral agents
☐	☐	Enhance the virulence of a pathogen or render a nonpathogen virulent
☐	☐	Increase transmissibility of a pathogen
☐	☐	Alter the host range of a pathogen
☐	☐	Enable evasion of diagnostic/detection modalities
☐	☐	Enable the weaponization of a biological agent or toxin
☐	☐	Any other potentially harmful combination of experiments and agents

ChIP-seq

Data deposition

- [ ] Confirm that both raw and final processed data have been deposited in a public database such as GEO.
- [ ] Confirm that you have deposited or provided access to graph files (e.g. BED files) for the called peaks.

Data access links<br>*May remain private before publication.*	*For "Initial submission" or "Revised version" documents, provide reviewer access links. For your "Final submission" document, provide a link to the deposited data.*
Files in database submission	*Provide a list of all files available in the database submission.*
Genome browser session<br>(e.g. UCSC)	*Provide a link to an anonymized genome browser session for "Initial submission" and "Revised version" documents only, to enable peer review. Write "no longer applicable" for "Final submission" documents.*

Methodology

Replicates	*Describe the experimental replicates, specifying number, type and replicate agreement.*
Sequencing depth	*Describe the sequencing depth for each experiment, providing the total number of reads, uniquely mapped reads, length of reads and whether they were paired- or single-end.*
Antibodies	*Describe the antibodies used for the ChIP-seq experiments; as applicable, provide supplier name, catalog number, clone name, and lot number.*
Peak calling parameters	*Specify the command line program and parameters used for read mapping and peak calling, including the ChIP, control and index files used.*
Data quality	*Describe the methods used to ensure data quality in full detail, including how many peaks are at FDR 5% and above 5-fold enrichment.*
Software	*Describe the software used to collect and analyze the ChIP-seq data. For custom code that has been deposited into a community repository, provide accession details.*

Flow Cytometry

Plots

Confirm that:

- [ ] The axis labels state the marker and fluorochrome used (e.g. CD4-FITC).
- [ ] The axis scales are clearly visible. Include numbers along axes only for bottom left plot of group (a 'group' is an analysis of identical markers).
- [ ] All plots are contour plots with outliers or pseudocolor plots.
- [ ] A numerical value for number of cells or percentage (with statistics) is provided.

Methodology

Sample preparation	*Describe the sample preparation, detailing the biological source of the cells and any tissue processing steps used.*
Instrument	*Identify the instrument used for data collection, specifying make and model number.*
Software	*Describe the software used to collect and analyze the flow cytometry data. For custom code that has been deposited into a community repository, provide accession details.*
Cell population abundance	*Describe the abundance of the relevant cell populations within post-sort fractions, providing details on the purity of the samples and how it was determined.*
Gating strategy	*Describe the gating strategy used for all relevant experiments, specifying the preliminary FSC/SSC gates of the starting cell population, indicating where boundaries between "positive" and "negative" staining cell populations are defined.*

- [ ] Tick this box to confirm that a figure exemplifying the gating strategy is provided in the Supplementary Information.

Magnetic resonance imaging

Experimental design

Design type	*Indicate task or resting state; event-related or block design.*

Design specifications

Specify the number of blocks, trials or experimental units per session and/or subject, and specify the length of each trial or block (if trials are blocked) and interval between trials.

Behavioral performance measures

State number and/or type of variables recorded (e.g. correct button press, response time) and what statistics were used to establish that the subjects were performing the task as expected (e.g. mean, range, and/or standard deviation across subjects).

Acquisition

Imaging type(s)

Specify: functional, structural, diffusion, perfusion.

Field strength

Specify in Tesla

Sequence & imaging parameters

Specify the pulse sequence type (gradient echo, spin echo, etc.), imaging type (EPI, spiral, etc.), field of view, matrix size, slice thickness, orientation and TE/TR/flip angle.

Area of acquisition

State whether a whole brain scan was used OR define the area of acquisition, describing how the region was determined.

Diffusion MRI ☐ Used ☐ Not used

Preprocessing

Preprocessing software

Provide detail on software version and revision number and on specific parameters (model/functions, brain extraction, segmentation, smoothing kernel size, etc.).

Normalization

If data were normalized/standardized, describe the approach(es): specify linear or non-linear and define image types used for transformation OR indicate that data were not normalized and explain rationale for lack of normalization.

Normalization template

Describe the template used for normalization/transformation, specifying subject space or group standardized space (e.g. original Talairach, MNI305, ICBM152) OR indicate that the data were not normalized.

Noise and artifact removal

Describe your procedure(s) for artifact and structured noise removal, specifying motion parameters, tissue signals and physiological signals (heart rate, respiration).

Volume censoring

Define your software and/or method and criteria for volume censoring, and state the extent of such censoring.

Statistical modeling & inference

Model type and settings

Specify type (mass univariate, multivariate, RSA, predictive, etc.) and describe essential details of the model at the first and second levels (e.g. fixed, random or mixed effects; drift or auto-correlation).

Effect(s) tested

Define precise effect in terms of the task or stimulus conditions instead of psychological concepts and indicate whether ANOVA or factorial designs were used.

Specify type of analysis: ☐ Whole brain ☐ ROI-based ☐ Both

Statistic type for inference
(See Eklund et al. 2016)

Specify voxel-wise or cluster-wise and report all relevant parameters for cluster-wise methods.

Correction

Describe the type of correction and how it is obtained for multiple comparisons (e.g. FWE, FDR, permutation or Monte Carlo).

Models & analysis

n/a	Involved in the study
☐	☐ Functional and/or effective connectivity
☐	☐ Graph analysis
☐	☐ Multivariate modeling or predictive analysis

Functional and/or effective connectivity

Report the measures of dependence used and the model details (e.g. Pearson correlation, partial correlation, mutual information).

Graph analysis

Report the dependent variable and connectivity measure, specifying weighted graph or binarized graph, subject- or group-level, and the global and/or node summaries used (e.g. clustering coefficient, efficiency, etc.).

Multivariate modeling and predictive analysis

Specify independent variables, features extraction and dimension reduction, model, training and evaluation metrics.
